# Advanced glycation end-products, measured as skin autofluorescence, associate with vascular stiffness in diabetic, pre-diabetic and normoglycemic individuals: a cross-sectional study

**DOI:** 10.1186/s12933-021-01296-5

**Published:** 2021-06-27

**Authors:** Anna Birukov, Rafael Cuadrat, Elli Polemiti, Fabian Eichelmann, Matthias B. Schulze

**Affiliations:** 1 Department of Molecular Epidemiology, German Institute of Human Nutrition Potsdam-Rehbrücke, Arthur-Scheunert-Allee 114-116, 14558 Nuthetal, Germany; 2 German Center for Diabetes Research (DZD), München-Neuherberg, Germany; 3 Institute of Nutritional Science, University of Potsdam, Nuthetal, Germany

**Keywords:** Advanced glycation end-products, AGE, Ankle-brachial index, Augmentation index, Prediabetes, Glycemia, Pulse wave velocity, Skin autofluorescence, Vascular stiffness

## Abstract

**Background:**

Advanced glycation end-products are proteins that become glycated after contact with sugars and are implicated in endothelial dysfunction and arterial stiffening. We aimed to investigate the relationships between advanced glycation end-products, measured as skin autofluorescence, and vascular stiffness in various glycemic strata.

**Methods:**

We performed a cross-sectional analysis within the European Prospective Investigation into Cancer and Nutrition (EPIC)-Potsdam cohort, comprising n = 3535 participants (median age 67 years, 60% women). Advanced glycation end-products were measured as skin autofluorescence with AGE-Reader™, vascular stiffness was measured as pulse wave velocity, augmentation index and ankle-brachial index with Vascular Explorer™. A subset of 1348 participants underwent an oral glucose tolerance test. Participants were sub-phenotyped into normoglycemic, prediabetes and diabetes groups. Associations between skin autofluorescence and various indices of vascular stiffness were assessed by multivariable regression analyses and were adjusted for age, sex, measures of adiposity and lifestyle, blood pressure, prevalent conditions, medication use and blood biomarkers.

**Results:**

Skin autofluorescence associated with pulse wave velocity, augmentation index and ankle-brachial index, adjusted beta coefficients (95% CI) per unit skin autofluorescence increase: 0.38 (0.21; 0.55) for carotid-femoral pulse wave velocity, 0.25 (0.14; 0.37) for aortic pulse wave velocity, 1.00 (0.29; 1.70) for aortic augmentation index, 4.12 (2.24; 6.00) for brachial augmentation index and − 0.04 (− 0.05; − 0.02) for ankle-brachial index. The associations were strongest in men, younger individuals and were consistent across all glycemic strata: for carotid-femoral pulse wave velocity 0.36 (0.12; 0.60) in normoglycemic, 0.33 (− 0.01; 0.67) in prediabetes and 0.45 (0.09; 0.80) in diabetes groups; with similar estimates for aortic pulse wave velocity. Augmentation index was associated with skin autofluorescence only in normoglycemic and diabetes groups. Ankle-brachial index inversely associated with skin autofluorescence across all sex, age and glycemic strata.

**Conclusions:**

Our findings indicate that advanced glycation end-products measured as skin autofluorescence might be involved in vascular stiffening independent of age and other cardiometabolic risk factors not only in individuals with diabetes but also in normoglycemic and prediabetic conditions. Skin autofluorescence might prove as a rapid and non-invasive method for assessment of macrovascular disease progression across all glycemic strata.

**Supplementary Information:**

The online version contains supplementary material available at 10.1186/s12933-021-01296-5.

## Background

Vascular stiffness is a pathophysiological process involving endothelial and vascular smooth muscle cells (VSMCs), extracellular matrix (ECM), perivascular adipose tissue and other integral components of the vascular wall [1]. Arterial stiffness independently predicts cardiovascular risk, causing isolated systolic hypertension and excessive penetration of pulse pressure into the microvasculature of target organs that operate at low vascular resistance, contributing to end-organ damage, and promoting left ventricular remodeling, dysfunction, and failure [2]. The underlying mechanisms of vascular stiffness are still incompletely understood, but accumulating evidence supports the involvement of pathogenic factors such as advanced glycation end-products (AGE) in its pathogenesis [3]. AGE are stable compounds that accumulate on long-lived proteins; they are formed by the reaction of proteins or lipids with aldose sugars and further molecular rearrangements. AGE formation is accelerated in hyperglycemia, renal failure, and inflammatory conditions [3]. AGE promote and exacerbate endothelial dysfunction [4] and functional arterial stiffening by reducing the phosphorylation status and expression of endothelial nitric oxide synthase (eNOS) [3, 5]. Moreover, AGE have been linked to structural arterial stiffening via crosslinking the collagen and elastin molecules, which results in an increase in the ECM area and VSMC phenotypic changes [3, 6]. AGE also activate Toll-like receptor 4/NF-κ light-chain enhancer of activated B cells (NF-κB) [3, 7], which contributes to the development of vascular inflammation. In recent years, skin autofluorescence (AF) emerged as a non-invasive marker of AGE accumulation in the skin tissue [8–10].

Human studies examining the associations between AGE and arterial stiffness focused primarily on high-risk patients with chronic disease, such as hypertension [11], end-stage renal disease [12], diabetes type 1 [13] and type 2 [9, 14]. As of now, the relationships between AGE and vascular stiffness in persons without cardiometabolic disease are equivocal [9, 15, 16]. There is also a paucity of insight in the interrelationships of AGE with glucose and HbA_1c_ levels relating to vascular stiffness. Vascular stiffness have been shown to increase with higher HbA_1c_ and glucose levels in diabetes patients [17, 18], however, the findings in normoglycemic persons are inconsistent [18, 19].

We hypothesized that AGE measured as skin AF are associated with vascular stiffness across all glycemic strata, and investigated these relationships in a cross-sectional sub-study (EPIC-DZD) nested within the population-based European Prospective Investigations into Cancer and Nutrition (EPIC) Potsdam cohort.

## Methods

### Study design and participants

We performed a cross-sectional analysis within the European Prospective Investigation into Cancer and Nutrition (EPIC) Potsdam Study, a population-based unselected longitudinal cohort from the municipality of Potsdam, Germany. At baseline, approx. 27,500 men and women were recruited between 1994 and 1998 [20]. Participants are followed up via questionnaires regarding incident diseases, all-cause mortality, dietary patterns, physical activity status, lifestyle and anthropometric measures. Of those participants who responded to the 6th wave of follow-up questionnaires between 2014 and 2016 (n = 15,424), n = 8517 were invited for a physical examination between 2014 and 2020 to collect data on nutritional and cardiovascular phenotyping and to identify risk factors for development and progression of chronic diseases, such as type 2 diabetes mellitus. Until 31 January 2020, 4370 participants were examined. The analytical sample comprised 3535 participants with available skin AF and vascular stiffness measurements, Additional file 1: Figure S1. All examinations were performed in quiet and temperature-controlled rooms by trained study nurses using standardized protocols. The study was conducted according to the Declaration of Helsinki and approved by the Ethical Committee of the State of Brandenburg, Germany. All participants provided written informed consent.

### Measurements of AGE

AGE accumulation in the skin was estimated with skin autofluorescence as measured with AGE-Reader (DiagnOptics Technologies B.V., Version SU 2.4.2.2, Groningen, The Netherlands). The technical description of the measurement is provided elsewhere [8, 9]. Skin AF was measured in seated position on the inner side of the right forearm below the elbow fold in three consecutive measurements, as described by the manufacturer. The mean of 3 measurements was used in the analyses. If a participant had tattoos, pigmentation, wounds or scars on the right forearm, measurements on the left forearm were performed instead.

### Measurements of vascular stiffness

Parameters of vascular stiffness (carotid-femoral and aortic pulse wave velocities [cfPWV and aoPWV], brachial and aortic augmentation indices [brAIx and aoAIx], ankle-brachial index [ABI]) were recorded and calculated by Vascular Explorer software (Enverdis GmbH, Jena, Germany). Vascular Explorer implements single-point, suprasystolic brachial oscillometry pulse wave analysis for the assessment of PWV and AIx, was validated against other oscillometric, photoplethysmographic and Doppler devices and deemed feasible in epidemiologic studies [21, 22]. Measurements were performed with appropriate arm and leg cuffs after at least 10 min resting in supine position. Pulse wave analysis, ankle and brachial blood pressures were automatically computed by software mediated analysis of photoplethysmographic signals from finger and toe and volume changes in the inflatable cuffs. The ABI was calculated by the software using the blood pressure readings in the lower and upper extremities. Participants were asked to refrain from speaking and advised to breathe calmly during the measurements. Exclusion criteria for this examination were amputations of limbs, open wounds at measurement sites, dialysis shunts, paralyses, lymph edema on arms or legs, bandages, or anti-embolism compression stockings which could not be removed.

### Measurements of glycemic and other laboratory parameters

Low-density lipoprotein (LDL), high-density lipoprotein (HDL) and total cholesterol, triglycerides and C-reactive protein (CRP) were measured in serum, HbA_1c_ in plasma EDTA. Blood samples were taken at random and immediately processed for further analyses. A three-point oral glucose tolerance test (OGTT) was performed on a subset of fasting participants (n = 1348). Exclusion criteria for OGTT were established diabetes, antidiabetic treatment, fasting plasma glucose (FPG) < 3.9 mmol/l or ≥ 11.1 mmol/l, or participant’s unwillingness to undergo the OGTT procedure. Participants were offered a 75 g oral glucose load, and fasting as well as 2-h plasma glucose concentrations were determined. All laboratory analyses were performed at the Institute for Medical Diagnostics Berlin-Potsdam using automated, standardized and quality-controlled assays.

### Statistical analyses

Characteristics of the study participants were evaluated according to the quintiles of skin AF. Missing data were not imputed, participants with missing data were excluded listwise. Right-skewed concentrations of HbA_1c_, triglycerides and CRP were normalized by log-transformation prior to any analyses. For associations between skin AF and vascular stiffness, linear and logistic multivariable regressions were constructed. Model 1 was adjusted for sex and age, model 2 was additionally adjusted for BMI, waist circumference, smoking status (three categories: non-smoker, former smoker, current smoker), recreational physical activity (biking and sports, h/week), systolic and diastolic blood pressure, pulse, prevalent conditions (prevalent heart failure, prior myocardial infarction or stroke), antihypertensive and lipid-lowering treatment, CRP, LDL-, HDL- and total cholesterol, triglycerides and HbA_1c_. Interactions were evaluated on multiplicative scale by creating cross product terms and assessing the alpha level. For the stratified analyses, abdominal adiposity was defined using sex-specific clinical cut-offs: in men > 102 cm, in women > 88 cm. In addition to the analyses on continuous measures of vascular stiffness, we defined vascular stiffness by two thresholds—cfPWV ≥ 10 m/s and cfPWV ≥ 12 m/s—as recommended by the ESC guidelines [23, 24]. The shape of association between skin AF with vascular stiffness was evaluated with restricted cubic splines with three knots at 5th, 50th and 95th percentiles. Median skin AF was used as the reference, non-linear trend in the splines was assessed with the Wald test.

Further, we evaluated whether the relationships between skin AF and vascular stiffness were modified by glycemic status. To this end, we categorized the participants according to their HbA_1c_ level and use of antidiabetic drugs in three groups: normoglycemic (HbA_1c_ < 5.7% and no antidiabetic treatment), prediabetes (6.5% > HbA_1c_ ≥ 5.7% and no antidiabetic treatment), diabetes (HbA_1c_ ≥ 6.5% or prevalent diabetes at EPIC-Potsdam baseline, or antidiabetic treatment), Additional file 1: Figure S1. In participants with available OGTT (n = 1348) we further characterized the prediabetes group based on several aspects of impaired glucose metabolism, such as impaired fasting glucose (IFG: 7 mmol/l > FPG ≥ 5.6 mmol/l), impaired glucose tolerance (IGT: 11.1 mmol/l > 2 h plasma glucose ≥ 7.8 mmol/l), abnormal HbA_1c_ (6.5% > HbA_1c_ ≥ 5.7%) or any abnormal glycemic value (IFG or IGT or abnormal HbA_1c_), Additional File 1: Figure S1.

A two-sided p < 0.05 denoted statistical significance. All statistical analyses were performed using SAS (Version 9.4, Enterprise Guide 7.1, SAS Institute Inc., Cary, NC, USA).

## Results

### Characteristics of study participants

**Fig. 1:**
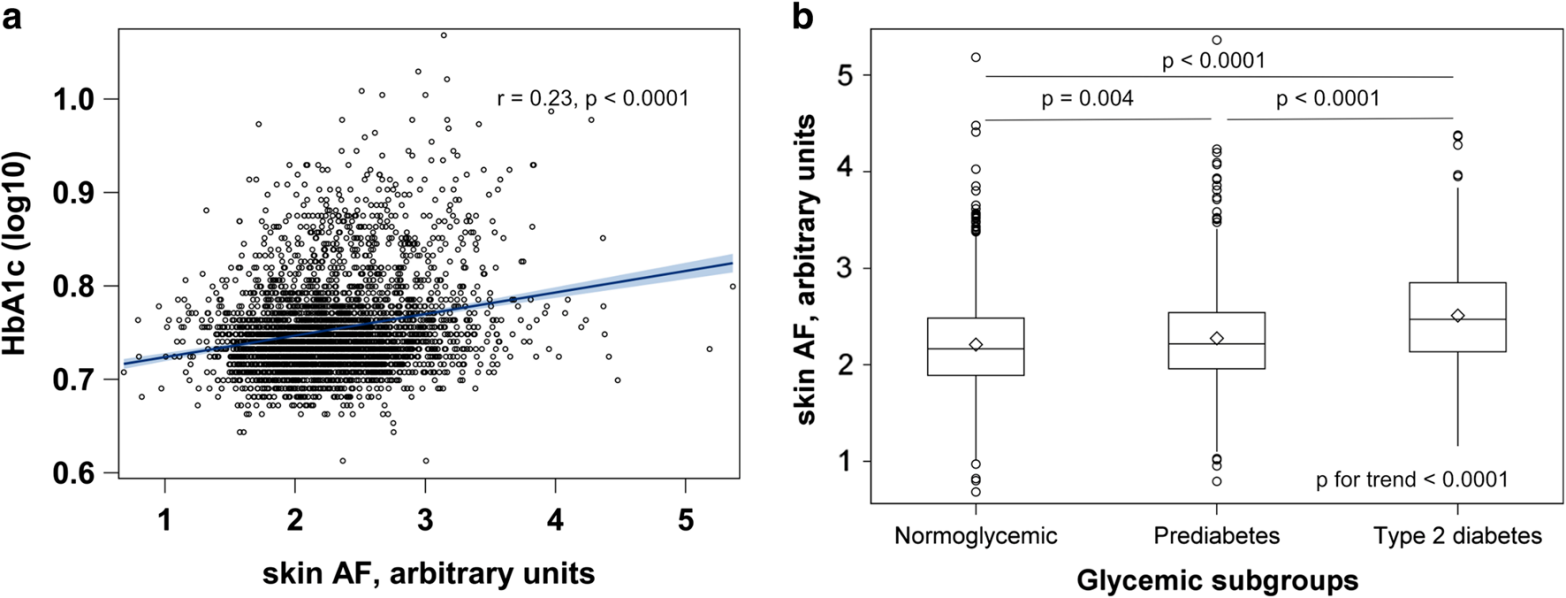
Associations between skin AF and glycemic status in the EPIC-DZD study. N = 3535. Glycemic status was defined as follows: normoglycemic (HbA1c < 5.7% and no antidiabetic treatment), prediabetes (6.5% > HbA1c ≥ 5.7% and no antidiabetic treatment), diabetes (antidiabetic treatment, HbA1c ≥ 6.5% or prevalent diabetes at EPIC-Potsdam baseline). Differences in medians of skin AF were compared with 1-way ANOVA with Tukey–Kramer’s correction for multiple testing. Linear trend in skin AF across glycemic strata was assessed with linear regression. *AF* autofluorescence, *HbA*_*1c*_ glycated hemoglobin 1C

Participants’ characteristics according to the quintiles of skin AF are shown in Table 1. Participants with higher skin AF values were on average older, more likely to be men, had higher BMI, waist circumference and blood pressure. The fraction of smokers, hypertensive individuals, participants with CVD, and individuals on antihypertensive, antidiabetic and lipid-lowering drugs increased across skin AF quintiles. Concentrations of CRP, triglycerides and creatinine also increased with higher skin AF, while LDL-, HDL- and total cholesterol decreased across skin AF quintiles. In unadjusted analyses, skin AF positively correlated with HbA_1c_, Fig. 1a and increased linearly across all glycemic strata, p < 0.0001 for trend, Fig. 1b. The median skin AF (IQR) was 2.17 (1.89–2.48) for normoglycemic, 2.22 (1.96–2.54) for prediabetes, 2.47 (1.14–2.85) for diabetes groups.

**Table 1:** Participants’ characteristics of the EPIC-DZD study according to quintiles of skin AF

Skin AF (arbitrary units)	1st quintile	2nd quintile	3rd quintile	4th quintile	5th quintile
	1.70 (0.22)	2.00 (0.12)	2.23 (0.12)	2.49 (0.15)	2.92 (0.39)
Sociodemographics					
Age, y	64.0 (12.0)	65.0 (12.0)	67.0 (11.0)	69.0 (12.0)	73.0 (11.0)
Women, n (%)	559 (79.1)	509 (71.9)	431 (61.1)	347 (49.1)	264 (37.3)
BMI, kg/m^2^	25.8 (5.44)	25.9 (5.85)	26.7 (5.44)	26.2 (5.65)	27.0 (5.44)
Waist circumference, cm	87.2 (17.3)	90.0 (19.0)	92.4 (18.3)	93.7 (18.2)	96.8 (16.3)
Obesity, n (%)	132 (18.7)	142 (20.1)	150 (21.3)	162 (19.5)	182 (25.7)
Recreational physical activity					
Biking, h/week	1.50 (4.00)	2.00 (5.00)	2.00 (5.00)	2.00 (5.00)	1.00 (4.00)
Sport, h/week	2.00 (2.83)	2.00 (3.00)	2.00 (3.00)	1.50 (3.00)	1.42 (3.00)
Smoking status					
Current smoker, n (%)	37 (5.2)	48 (6.8)	49 (6.9)	72 (10.2)	92 (13.0)
Former smoker, n (%)	220 (31.1)	250 (35.3)	300 (42.5)	285 (40.3)	325 (46.0)
SBP, mmHg	137 (24.0)	137 (25.0)	139 (24.0)	141 (24.0)	141 (24.0)
DBP, mmHg	80.0 (12.0)	81.0 (13.0)	81.0 (13.0)	80.0 (13.0)	78.0 (14.0)
Pulse, beats/min	67.0 (12.0)	67.0 (12.0)	66.0 (13.0)	66.0 (14.0)	67.0 (14.0)
Antidiabetic treatment, n (%)	53 (7.5)	71 (10.0)	95 (13.5)	124 (17.5)	204 (28.9)
Lipid-lowering treatment, n (%)	138 (19.5)	146 (20.6)	189 (26.8)	188 (26.6)	290 (41.0)
Prevalent hypertension, n (%)	499 (70.6)	500 (70.6)	544 (77.1)	553 (78.2)	614 (86.9)
Antihypertensive treatment, n (%)	324 (45.8)	335 (47.3)	381 (54.0)	393 (55.6)	476 (67.3)
Prevalent HF, n (%)	5 (0.7)	4 (0.6)	8 (1.1)	4 (0.6)	12 (1.7)
Prior stroke, n (%)	11 (1.6)	17 (2.4)	11 (1.6)	10 (1.4)	17 (2.4)
Prior MI, n (%)	2 (0.3)	7 (1.0)	9 (1.3)	19 (2.7)	25 (3.5)
Biomarkers					
HbA_1c_, %	5.50 (0.50)	5.50 (0.40)	5.50 (0.50)	5.50 (0.60)	5.70 (0.80)
HbA_1c_, mmol/mol	36.6 (3.33)	36.6 (2.66)	36.6 (3.33)	36.6 (4.08)	38.8 (5.45)
Total cholesterol, mmol/l	5.67 (1.39)	5.58 (1.32)	5.49 (1.62)	5.44 (1.60)	5.16 (1.62)
Total cholesterol, mmol/l^a^	5.92 (1.35)	5.86 (1.24)	5.81 (1.35)	5.73 (1.26)	5.63 (1.25)
HDL-cholesterol, mmol/l	1.63 (0.55)	1.57 (0.59)	1.51 (0.50)	1.49 (0.55)	1.41 (0.53)
HDL-cholesterol, mmol/l^a^	1.66 (0.53)	1.60 (0.58)	1.57 (0.49)	1.53 (0.55)	1.50 (0.53)
LDL-cholesterol, mmol/l	3.50 (1.30)	3.50 (1.20)	3.40 (1.40)	3.40 (1.5)	3.10 (1.60)
LDL-cholesterol, mmol/l^a^	3.78 (1.10)	3.70 (1.15)	3.60 (1.12)	3.68 (1.10)	3.50 (1.00)
Triglycerides, mmol/l	1.28 (0.84)	1.29 (0.88)	1.37 (0.85)	1.33 (0.93)	1.40 (1.12)
CRP, mg/l	1.30 (1.90)	1.40 (2.00)	1.40 (2.00)	1.50 (2.20)	1.60 (2.40)
Creatinine, µmol/l	69.0 (18.0)	69.0 (17.0)	72.0 (18.0)	76.0 (21.0)	79.0 (22.0)

N = 3535. Hypertension was defined as self-reported hypertension, or use of antihypertensive medication, or systolic blood pressure ≥ 140 mmHg, or diastolic blood pressure ≥ 90 mmHg during blood pressure measurement in the study center. Obesity was defined as BMI ≥ 30 kg/m^2^

*AF* autofluorescence, *EPIC-DZD* Sub-study of European Prospective Investigations into Cancer and Nutrition, *BMI* body mass index, *SBP* systolic blood pressure, *DBP* diastolic blood pressure, *HbA*_*1c*_ hemoglobin A1c, *HDL* high density lipoprotein, *LDL* low density lipoprotein, *CRP* reactive protein C

^a^Blood lipids adjusted for lipid-lowering treatment. Data are presented as median (IQR) or number (%), as appropriate

### Relationships between skin AF and vascular stiffness in the EPIC-DZD study, n = 3535

In the adjusted models, skin AF positively associated with PWV, AIx and negatively with ABI, Table 2. In a comparative analysis with standardized beta coefficients, skin AF showed a stronger association with PWV and ABI than many other classic cardiometabolic risk factors, including age, waist circumference, HbA_1c_ and CRP, Additional file 1: Table S1. In its relation to AIx, effect estimates of skin AF were higher than those of HbA_1c_, CRP, cholesterol, triglycerides, Additional file 1: Table S1.

**Table 2:** Multivariable-adjusted associations between skin AF and continuous measures of vascular stiffness in the EPIC-DZD study

	Beta coefficients (95% CI) for skin AF, arbitrary units				
	cfPWV, m/s	aoPWV, m/s	aoAIx, %	brAIx, %	ABI
Model 1	0.41 (0.24; 0.58)	0.27 (0.16; 0.39)	0.28 (− 0.46; 1.02)	1.49 (− 0.63; 3.62)	− 0.04 (− 0.05; − 0.03)
Model 2	0.38 (0.21; 0.55)	0.25 (0.14; 0.37)	1.00 (0.29; 1.70)	4.12 (2.24; 6.00)	− 0.04 (− 0.05; − 0.02)

N = 3535. Model 1 was adjusted for age and sex. Model 2 was additionally adjusted for BMI, waist circumference, smoking status (three categories: non-smoker, former smoker, current smoker), recreational physical activity (biking and sports, h/week), systolic and diastolic blood pressure, pulse, prevalent conditions (prevalent heart failure, prior myocardial infarction or stroke), antihypertensive and lipid-lowering treatment, CRP, LDL-, HDL- and total cholesterol, triglycerides and HbA_1c_

*EPIC-DZD* Sub-study of European Prospective Investigations into Cancer and Nutrition, *AF* autofluorescence, *PWV* pulse wave velocity, *AIx* augmentation index, *ABI* ankle-brachial index, *ao* aortic, *br* brachial, *cf* carotid-femoral

**Fig. 2:**
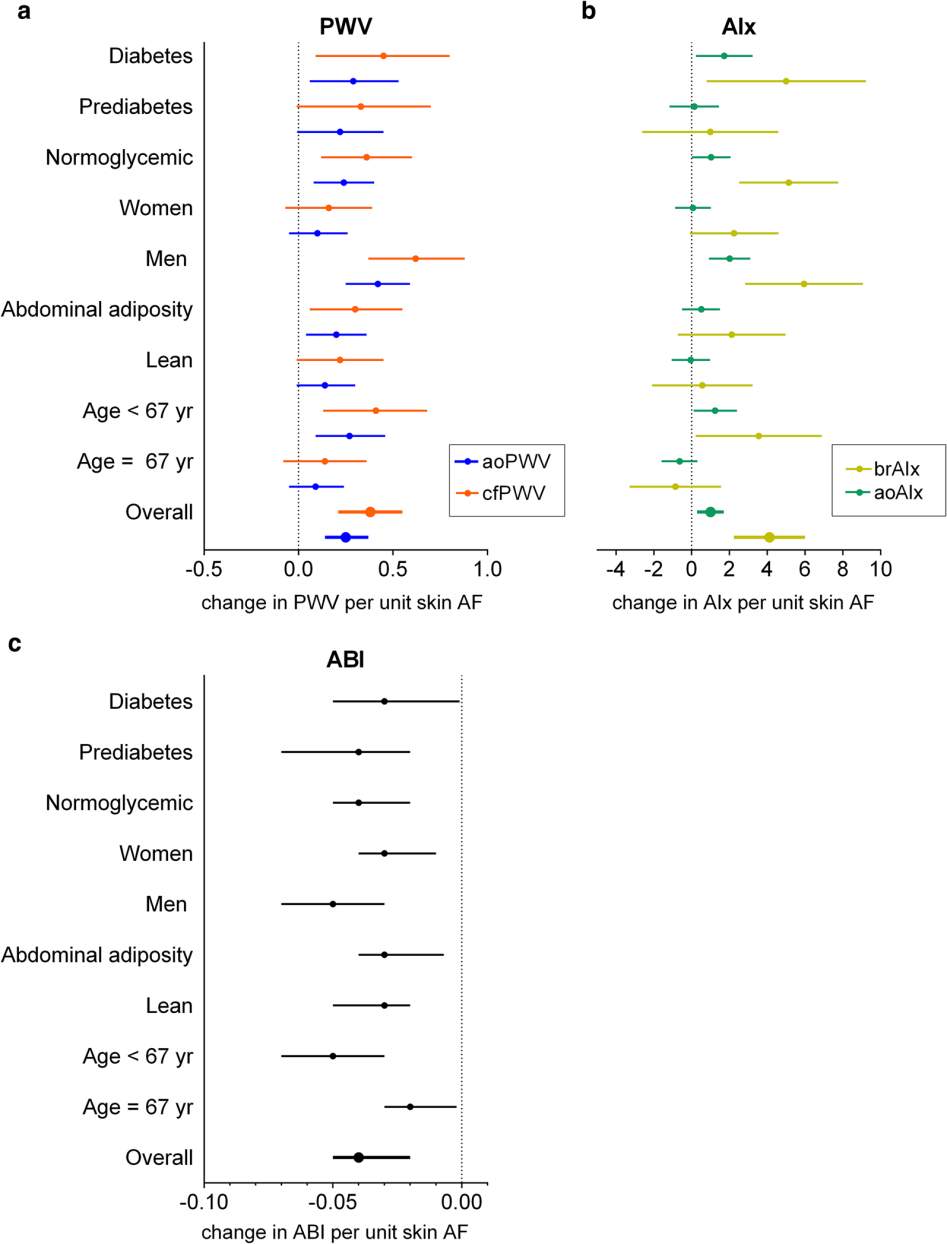
Adjusted associations between skin AF and vascular stiffness in the EPIC-DZD study. Adjusted associations between skin AF and parameters of vascular stiffness (**a** PWV, **b** AIx, **c** ABI) in the EPIC-DZD study, stratified by sex, median age, abdominal adiposity and glycemic status. Changes in vascular stiffness are per 1 unit increase in skin AF. Analyses were performed on fully adjusted model 2. p values for interaction terms: cfPWV: skin AF with sex, p = 0.002, with age, p = 0.07, with waist circumference, p = 0.02, with glycemic status, p = 0.77; aoPWV: skin AF with sex, p = 0.002, with age, p = 0.07, with waist circumference, p = 0.02, with glycemic status, p = 0.78; aoAIx: skin AF with sex, p = 0.0008, with age, p = 0.001, with waist circumference, p = 0.44, with glycemic status, p = 0.99; brAIx with sex, p = 0.006, with age, p = 0.004, with waist circumference, p = 0.67, with glycemic status, p = 0.37; ABI: skin AF with sex, p = 0.10, with age, p = 0.01, with waist circumference, p = 0.40, with glycemic status, p = 0.58. Analyses were adjusted for sex and age, BMI, waist circumference, smoking status (three categories: non-smoker, former smoker, current smoker), recreational physical activity (biking and sports, h/week), systolic and diastolic blood pressure, pulse, prevalent conditions (prevalent heart failure, prior myocardial infarction or stroke), antihypertensive and lipid-lowering treatment, CRP, LDL-, HDL- and total cholesterol, triglycerides and HbA1c. *EPIC-DZD* Sub-study of European Prospective Investigations into Cancer and Nutrition, *AF* autofluorescence, *PWV* pulse wave velocity, *AIx* augmentation index, *ABI* ankle-brachial index, *ao* aortic, *br* brachial, *cf* carotid-femoral

Because there was evidence for statistically significant interactions of skin AF with sex and age in relation to PWV and AIx, all analyses were stratified by sex and median age (67 years), Fig. 2. Stratified analyses revealed that the associations between skin AF and PWV, AIx were present only in men. One unit increase in skin AF was associated with 0.63 (95% CI 0.38; 0.89) m/s increase in cfPWV in men compared with 0.16 (− 0.06; 0.39) in women, with similar estimates for aoPWV, Fig. 2a. For aoAIx, the estimates were 1.98 (0.89; 3.07) in men and 0.02 (− 0.92; 0.96) in women, with a similar pattern for brAIx, Fig. 2b. Moreover, when stratified for median age, the associations between skin AF and vascular stiffness were markedly stronger in individuals < 67 years (for instance, cfPWV: 0.41 (0.14; 0.68) compared with 0.15 (− 0.07; 0.37) in individuals ≥ 67 years, brAIx: 3.53 (0.20; 6.86) compared with − 0.95 (− 3.36; 1.46)), Fig. 2a, b. Even though there was a significant interaction between skin AF and waist circumference (p = 0.02), the effect estimates in leaner individuals and individuals with abdominal adiposity were consistent, Fig. 2a, b. When we examined the relationships between skin AF and PWV across categories of glycemic strata defined by HbA_1c_ levels and antidiabetic treatment, the effect estimates were consistent across all glycemic strata: for cfPWV 0.36 (0.12; 0.60) in normoglycemic, 0.33 (− 0.01; 0.67) in prediabetes and 0.45 (0.09; 0.80) in diabetes groups; a similar direction was observed for aoPWV. AIx was associated with skin AF only in normoglycemic (1.03 (0.008; 2.05) for aoAIx and 5.14 (2.52; 7.76) for brAIx) and diabetes groups (1.72 (0.23; 3.22) for aoAIx and 5.00 (0.79; 9.22) for brAIx), but not in prediabetes group, Fig. 2a, b. ABI inversely associated with skin AF across all sex, age, obesity and glycemic strata, though stronger associations were present in men and younger individuals (Fig. 2c).

**Fig. 3:**
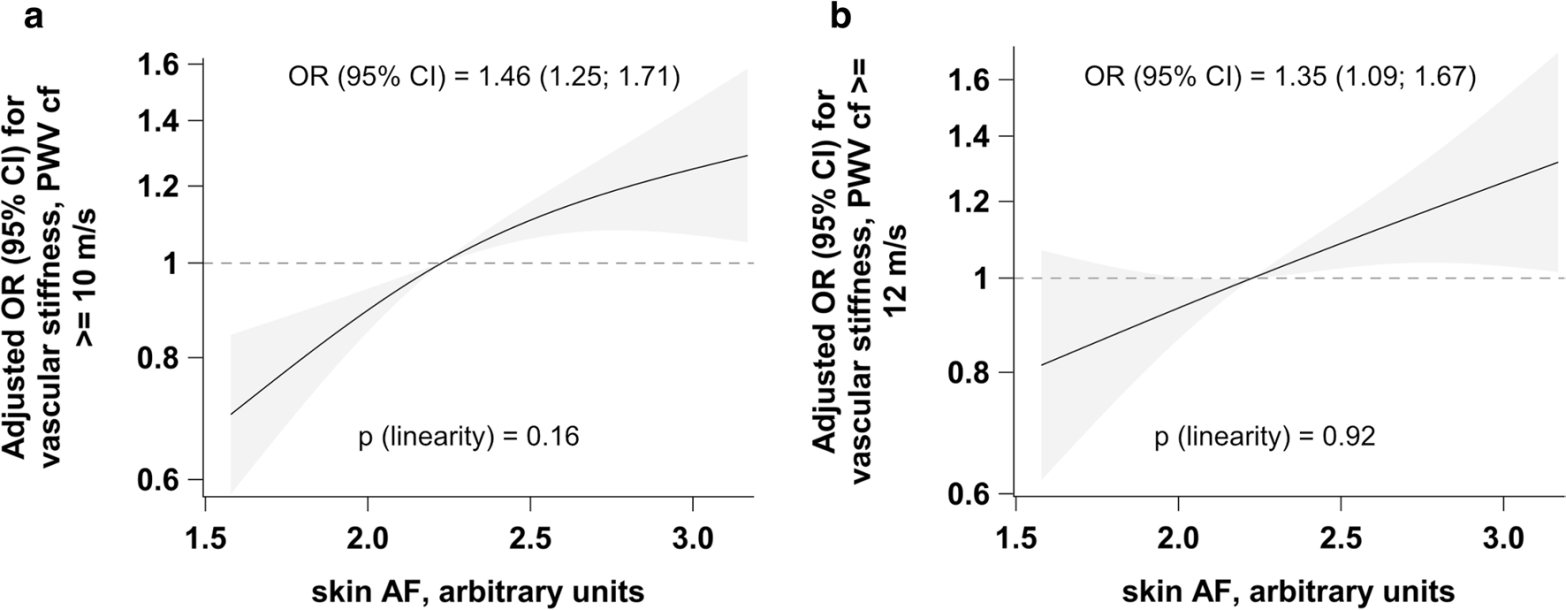
Adjusted odds ratios for relationships between skin AF and vascular stiffness. Adjusted associations between skin AF and vascular stiffness defined as cfPWV ≥ 10 m/s (**a**) and cfPWV ≥ 12 m/s (**b**). Linearity was assessed with Wald test from restricted cubic splines, adjusted ORs are reported from logistic regression and per 1 unit increase in skin AF. Analyses were adjusted for sex and age, BMI, waist circumference, smoking status (three categories: non-smoker, former smoker, current smoker), recreational physical activity (biking and sports, h/week), systolic and diastolic blood pressure, pulse, prevalent conditions (prevalent heart failure, prior myocardial infarction or stroke), antihypertensive and lipid-lowering treatment, CRP, LDL-, HDL- and total cholesterol, triglycerides and HbA1c. *AF* autofluorescence, *cfPWV* carotid-femoral pulse wave velocity, *OR* odds ratio, *CI* confidence interval

One unit increase in skin AF was associated with 46% higher OR for vascular stiffness when vascular stiffness was defined with a threshold of cfPWV ≥ 10 m/s (Fig. 3a) and 35% higher OR when vascular stiffness was defined with a threshold of cfPWV ≥ 12 m/s (Fig. 3b). No violation of linearity could be observed in the corresponding splines (Fig. 3).

### Relationships between skin AF and vascular stiffness across glycemic strata in the OGTT subset, n = 1348

Similar patterns of associations could be observed for glycemic strata defined by either FPG or as “any abnormal glycemic parameter” on the one hand and those strata defined by either HbA_1c_ or 2 h plasma glucose on the other hand. The associations of skin AF with PWV and brAIx appeared to be much stronger in the prediabetes groups compared to normoglycemic groups when those groups were defined based on FPG or as “any abnormal glycemic parameter”, Table 3. In contrast, the associations with PWV were not substantially different between normoglycemic and prediabetes groups in the strata defined by HbA_1c_ and 2 h plasma glucose levels. However, none of the interactions of skin AF with glycemic status in the OGTT subset was statistically significant. The estimates for ABI were consistent across all glycemic strata and definitions, with the exception of prediabetes group defined according to 2 h plasma glucose levels, Table 3.

**Table 3:** Associations between skin AF and continuous measures of vascular stiffness according to glycemic strata in the OGTT subset

	Beta coefficients (95% CI) for skin AF, arbitrary units				
	cfPWV, m/s	aoPWV, m/s	aoAIx, %	brAIx, %	ABI
Glycemic status according to any abnormal glycemic value					
Normoglycemic, n = 455	0.41 (− 0.09; 0.92)	0.27 (− 0.06; 0.61)	0.44 (− 1.59; 2.48)	0.08 (− 5.70; 5.87)	− 0.02 (− 0.06; 0.02)
Prediabetes, n = 795	0.85 (0.44; 1.26)	0.57 (0.29; 0.84)	1.74 (− 0.0002; 3.48)	8.02 (3.43; 12.62)	− 0.02 (− 0.05; 0.007)
Glycemic status according to HbA_1c_					
Normoglycemic, n = 994	0.62 (0.26; 0.98)	0.41 (0.17; 0.65)	1.35 (− 0.16; 2.87)	4.69 (0.64; 8.74)	− 0.03 (− 0.05; − 0.001)
Prediabetes, n = 338	0.78 (0.16; 1.40)	0.53 (0.11; 0.94)	0.94 (− 1.38; 3.27)	5.42 (− 0.93; 11.77)	− 0.01 (− 0.06; 0.03)
Glycemic status according to FPG					
Normoglycemic, n = 573	0.47 (0.03; 0.92)	0.31 (0.01; 0.61)	0.22 (− 1.59; 2.03)	1.60 (− 3.54; 6.73)	− 0.01 (− 0.04; 0.02)
Prediabetes (IFG), n = 709	0.93 (0.48; 1.38)	0.62 (0.32; 0.92)	2.23 (0.33; 4.14)	8.85 (3.90; 13.79)	− 0.03 (− 0.06; 0.003)
Glycemic status according to 2 h plasma glucose					
Normoglycemic, n = 1079	0.68 (0.34; 1.02)	0.45 (0.22; 0.68)	1.08 (− 0.35; 2.51)	4.19 (0.33; 8.06)	− 0.03 (− 0.05; − 0.008)
Prediabetes (IGT), n = 208	0.74 (− 0.08; 1.56)	0.49 (− 0.06; 1.04)	2.29 (− 0.77; 5.35)	7.98 (− 0.27; 16.22)	0.03 (− 0.03; 0.09)

N = 1348. Analyses were performed on fully adjusted model 2

Glycemic status was defined as follows:

According to HbA_1c_: normoglycemic, HbA_1c_ < 5.7%; prediabetes, 6.5% > HbA_1c_ ≥ 5.7%

According to FPG: normoglycemic, FPG < 5.6 mmol/l; prediabetes (IFG), 7 mmol/l > FPG ≥ 5.6 mmol/l

According to 2 h plasma glucose: normoglycemic, 2 h plasma glucose < 7.8 mmol/l; prediabetes (IGT), 11.1 mmol/l > 2 h plasma glucose ≥ 7.8 mmol/l

According to any abnormal glycemic parameter: normoglycemic, all three criteria fulfilled: HbA1c < 5.7% and FPG < 5.6 mmol/l and 2 h plasma glucose < 7.8 mmol/l; prediabetes, 6.5% > HbA_1c_ ≥ 5.7% or IFG or IGT

*AF* autofluorescence, *OGTT* oral glucose tolerance test, *PWV* pulse wave velocity, *AIx* augmentation index, *ABI* ankle-brachial index, *ao* aortic, *br* brachial, *cf* carotid-femoral, *HbA*_*1c*_ glycated hemoglobin 1c, *FPG* fasting plasma glucose, *IFG* impaired fasting glucose, *IGT* impaired glucose tolerance

## Discussion

This study demonstrates that AGE, measured as skin AF, were robustly associated with measures of arterial stiffness, such as PWV, AIx and ABI, independent of potential cardiometabolic confounders and glycemic status of the participants. The associations appeared to be more pronounced in men and younger individuals. Furthermore, the relationships of PWV and ABI with skin AFseemed to be stronger than with several other classic risk factors for atherosclerosis such as CRP, HbA_1c_, and even age.

Our findings are consistent with earlier studies investigating the associations between AGE, CVD, atherosclerosis and arterial stiffness in diabetes patients [9, 13, 14, 25]. However, in normoglycemic individuals, previous studies were inconclusive [9, 15, 16]. The discrepancies in these studies can be probably attributed to smaller sample size, measurement error, distinct measurements of vascular stiffness (PWV, AIx, pulse pressure, etc.) and AGE (plasma or serum measurements of specific AGE agents, skin autofluorescence) and differences in the composition (age, race, sex, etc.) of study populations. We demonstrate that the relationships between skin AF and vascular stiffness are not restricted to diabetic and prediabetic groups but are also present in normoglycemic populations. Mechanistic studies suggest that AGE promote endothelial dysfunction resulting in vasodilatory impairment [26], and diabetic and healthy individuals are on the whole equally vulnerable to the deleterious effects of acute AGE load on vascular endothelium [4]. Both endogenous and exogenous AGE amplify the expression and release of vascular cell adhesion molecule-1 [27, 28], increase production of reactive oxygen species and decrease NO bioavailability [3, 26], directly impacting endothelial cells and contributing to functional arterial stiffness. AGE modify ECM proteins in the vascular wall and may lead to the formation of intermolecular and intramolecular crosslinks with collagen and elastin, triggering structural arterial stiffness [3]. As a result of glycation, modification of lipoprotein promotes foam cell formation. Interestingly, in the EPIC-Potsdam cohort, HDL-, LDL- and total cholesterol all decreased across skin AF quintiles, even after adjustment for lipid-lowering treatment.

Our study is the first to evaluate the relationships between skin AF and several aspects of vascular stiffening—reflected by PWV, AIx and ABI—across all glycemic strata and in one setting. While aortic and carotid-femoral PVW is a proxy for large artery stiffness (arteriosclerosis), AIx was classically deemed a measure of wave reflection intended to quantify the deleterious effect of systolic wave reflection on cardiac workload, though its validity as a proxy for wave reflection magnitude has been questioned in recent studies [29]. ABI is an indicator of peripheral artery disease (PAD) progression, with most commonly used threshold ≤ 0.90 for diagnosis of PAD. Congruent with our findings that skin AF inversely associated with ABI, in a smaller clinical study performed in diabetes patients [30], plasma AGE inversely associated with ABI, indicating that AGE might contribute to the development of atherosclerosis in the below-the-knee peripheral artery tree both in diabetic and normoglycemic populations.

We further observed an indication that, though the direction of the associations between skin AF and vascular stiffness was the same across all glycemic strata, the strength of the relations may vary quite remarkably between normoglycemic and prediabetes participants, depending on the definition of prediabetes. Associations between skin AF and vascular stiffness tended to be stronger in prediabetes groups based on FPG or defined as any abnormal glycemic value compared to normoglycemic groups, whereas the relationships were not markedly different when prediabetes was based on HbA_1c_ or 2 h plasma glucose levels. This finding is supported by a recent Mendelian randomization (MR) study investigating causal effects of prediabetes on major diabetes-related complications, including coronary artery disease (CAD) [31]. Intriguingly, while FPG concentrations were causally related to CAD with a 26% higher odds of CAD per mmol/L increase in fasting glucose, no causal relationship between genetically elevated HbA_1c_ levels and diabetes complications could be detected in that study. FPG has been shown to be causally related to vascular stiffness independent of genetically elevated risk of type 2 diabetes in one study [32] and to subclinical atherosclerosis in individuals without diabetes in another MR study [33], although no other glycemic parameters except FPG were evaluated there. Moreover, accumulating evidence suggests that prediabetes stages defined by FPG (IFG) and 2 h plasma glucose (IGT) might have different pathophysiologies and consequences, thus representing fundamentally different metabolic conditions [34, 35]. IFG is characterized by reduced hepatic insulin sensitivity, stationary beta cell dysfunction and/or chronic low beta cell mass, altered glucagon-likepeptide-1 secretion and inappropriately elevated glucagon secretion. In contrast, IGT includes reduced peripheral insulin sensitivity, near-normal hepatic insulin sensitivity, progressive loss of beta cell function, reduced secretion of glucose-dependent insulinotropic polypeptide and inappropriately elevated glucagon secretion [34, 35]. A better understanding of the etiology and pathophysiological consequences of various prediabetic stages is the first step towards precision medicine in diabetes.

Altogether, these and previously reported data support the view that AGE might be involved in pathophysiological processes affecting vascular integrity and function, and preventive strategies targeting AGE accumulation, inhibiting AGE formation and interaction of AGE with their corresponding receptors (RAGE) appear as promising approaches to alleviate the AGE burden on the progression of vascular stiffness. Inhibition of AGE formation by aminoguanidine was shown to improve arterial compliance in older humans with vascular stiffening [36]. Recently, DNA aptamers targeting AGE–RAGE interaction have been developed, yielding encouraging results in experimental studies [37]. The use of alagebrium, an AGE-breaker, was inversely correlated with plasma matrix metalloproteinase-9 and type 1 collagen, suggesting that this drug could also reduce the structural arterial stiffness [38]. Finally, lifestyle interventions such as smoking secession and dietary AGE restriction could be tested to reduce the chronic impact of exogenous AGE on the vasculature [39].

Our study benefited from a large sample size in a low-risk setting, nested within population-based EPIC-Potsdam study. We assessed different measures of arterial stiffness, which allowed us to perform comparative analyses and not to restrict our analyses to the relationships with PWV only. Exposures, outcomes and various cardiometabolic confounders were measured by trained staff using standardized protocols, and the study personnel were unaware of specific research questions of this study, minimizing observer and reporting bias. Finally, a substantial number of participants underwent an OGTT, facilitating a refined characterization of the relationships between AGE and vascular stiffness in various glycemic strata with differing disease progression. Caveats include cross-sectional study design, and because of observational nature of our study, a residual confounding cannot be ruled out. Our study population was primarily of older individuals of European ancestry, and the findings need to be confirmed in other ethnicities and age groups. The accumulation of AGE is a general feature of the aging tissue due to glycation and oxidation reactions. However, we adjusted all analyses for age, thus, this factor is less likely to confound the results of this study. Further, AGE were measured only in skin and not e.g. in plasma. Consequently, it was not possible to identify which AGE agent was driving the observed relationships with vascular stiffness. We also could not study the interrelationships of skin-deposed AGE with circulating AGE and soluble receptors for AGE (sRAGE) regarding vascular stiffening. Mayer et al. indicated that the ratio of skin AF and sRAGE might be a more sensitive parameter for the assessment of annual changes in aortic PVW than skin AF or circulating AGE, because only this ratio remained significant in the stepwise regression model [40]. However, in another study in patients with aortic stenosis, the ratio between valvular AGE and plasma sRAGE was not statistically associated with the disease severity parameters such as aortic valve area in multivariable models, while valvular (tissue) AGE were significantly associated [41]. In comparative studies, the correlation between plasma and skin AGE was fairly moderate to good [42]. However, circulating AGE are more prone to fluctuations due to dietary intake and excretion and therefore may not accurately reflect the long-term AGE accumulation in tissues. Skin AF can be used in subjects with a skin pigmentation up to Fitzpatrick type V [10]. It utilizes auto-fluorescent properties which certain dermal AGE such as pentosidine possess and has been validated with the AGE content measured in skin biopsies [8]. Meerwaldt et al. showed a strong correlation between skin AF measured with AGE-Reader and the fluorescent AGE CLF and pentosidine as well as the non-fluorescent AGE CML and Nε-carboxyethyl-lysine in the dermal layer of the skin, which were obtained from skin biopsies [8]. Moreover, Hofmann et al. found that skin AF strongly correlated with collagen fractions isolated from vein graft material by proteolysis and collagenase digestion and quantified by hydroxyproline assay, and both parameters correlated with PWV [43].

Since the AGE Reader™ uses light to detect AGE, the measurements in individuals with a very dark skin are difficult, due to absorption of both the incoming light and the fluorescent light. However, EPIC-DZD participants were exclusively of central European origin with light skin. Another limitation of the device is a potential interference of skin cream with skin AF measurements. Especially self-browning and sun blocker creams could block the incident light and cause unreliable skin AF measurements. Of all participants, who underwent AGE measurements, only 13 participants reported using self-browning, sun blocker or other skin cream, and the median AGE values in these participants were not different from those, who did not use any skin cream. Skin AF might prove useful as a rapid, non-invasive and cost-effective method to evaluate tissue AGE accumulation in interventional studies.

## Conclusions

In conclusion, in community-dwelling older adults, AGE measured by skin AF associated with measures of arteriosclerosis, wave reflection and peripheral arterial disease and appeared as an important contributor to the development of macrovascular dysfunction independent of cardiometabolic confounders. The observed relationships were strongest in men and younger individuals and persisted across all glycemic strata.

## Supplementary Information

**Additional file 1. Figure S1**. Flowchart of inclusion. **Table S1. **Mutually-adjusted associations between skin AF and vascular stiffness in the EPIC-DZD Study.

## Data Availability

The datasets analyzed during the current study are not publicly available due to data protection regulations. In accordance with German Federal and State data protection regulations, epidemiological data analyses of EPIC-DZD may be initiated upon an informal inquiry addressed to the secretariate of the Human Study Center (Office.HSZ@dife.de). Each request will then have to pass a formal process of application and review by the respective PI and a scientific board.

## Abbreviations

ABI: Ankle-brachial index
AF: Autofluorescence
AGE: Advanced glycation end-products
AIx: Augmentation index
ao: Aortic
br: Brachial
BMI: Body mass index
CAD: Coronary artery disease
cf: Carotid-femoral
CI: Confidence interval
CRP: C-reactive protein
CVD: Cardiovascular disease
DBP: Diastolic blood pressure
ECM: Extracellular matrix
eNOS: Endothelial nitric oxide synthase
EPIC: European Prospective Investigation into Cancer and Nutrition study
ESC: European Society of Cardiology
FPG: Fasting plasma glucose
HbA_1c_: Hemoglobin A1c
HDL: High-density lipoprotein cholesterol
IFG: Impaired fasting glucose
IGT: Impaired glucose tolerance
IQR: Interquartile range
LDL: Low-density lipoprotein cholesterol
MR: Mendelian randomization
NF-κB: NF-κ light-chain enhancer of activated B cells
NO: Nitric oxide
OGTT: Oral glucose tolerance test
OR: Odds ratio
PAD: Peripheral artery disease
PWV: Pulse wave velocity
SBP: Systolic blood pressure
VSMC: Vascular smooth muscle cells
