# Supplementary material for: Advanced glycation end-products, measured as skin autofluorescence, associate with vascular stiffness in diabetic, pre-diabetic and normoglycemic individuals: a cross-sectional study
Source: Cardiovasc Diabetol. 2021 Jun 27;20:110. doi: 10.1186/s12933-021-01296-5 (PMC8236143; doi:10.1186/s12933-021-01296-5)
Supplement: Supplementary file 1 — Additional file 1. Figure S1. Flowchart of inclusion. Table S1. Mutually-adjusted associations between skin AF and vascular stiffness in the EPIC-DZD Study. [file 12933_2021_1296_MOESM1_ESM.docx]

**Additional File 1**

**Figure S1**. Flowchart of inclusion.

**Glycemic status according to HbA1c**

Normoglycemic, n=2,088

Prediabetes, n=805

Diabetes, n=642

**Glycemic status according to HbA_1c_**

Normoglycemic, n=994

Prediabetes, n=338

Diabetes, n=16

**Glycemic status according to FPG**

Normoglycemic, n=573

Prediabetes (IFG), n=709

Diabetes, n=66

**Glycemic status according to 2h plasma glucose**

Normoglycemic, n=1,079

Prediabetes (IGT), n=208

Diabetes, n=61

**Glycemic status according to any abnormal glycemic value**

Normoglycemic, n=455

Prediabetes, n=795

Diabetes, n=98

**Exclusions**

n=835

Missing AF measurement, n=446

Missing vascular stiffness measurement, n=219

Missing information on confounders, n=170

**Did not accept invitation**

n=4,147

**Invitation**

n=8,517

**EPIC-DZD**

n=4,370

**OGTT subset**

n=1,348

**Cohort available for main analyses**

n=3,535

**Responded to the 6^th^ follow-up questionnaires (2014-2020)**

n=15,424

**EPIC-Potsdam**

n=27,548

Glycemic status was defined as follows:

according to HbA_1c_: normoglycemic, HbA_1c_ <5.7%; prediabetes, 6.5%> HbA_1c_ ≥5.7%; diabetes, HbA_1c_ ≥6.5%;

according to FPG: normoglycemic, FPG <5.6 mmol/l; prediabetes (IFG), 7 mmol/l> FPG ≥5.6 mmol/l; diabetes, FPG ≥7 mmol/l;

according to 2h plasma glucose: normoglycemic, 2h plasma glucose <7.8 mmol/l; prediabetes (IGT), 11.1 mmol/l> 2h plasma glucose ≥7.8 mmol/l; diabetes, 2h plasma glucose ≥11.1 mmol/l;

according to any abnormal glycemic parameter: normoglycemic, all 3 criteria fulfilled: HbA_1c_ <5.7% and FPG <5.6 mmol/l and 2h plasma glucose <7.8 mmol/l; prediabetes, 6.5%> HbA_1c_ ≥5.7% or IFG or IGT; diabetes, HbA_1c_ ≥6.5% or FPG ≥7 mmol/l or 2h plasma glucose ≥11.1 mmol/l.

**Table S1.** Mutually-adjusted associations between skin AF and vascular stiffness in the EPIC-DZD Study.

| **Predictors** | **Beta coefficients (95% CI)** | | | | |
| --- | --- | --- | --- | --- | --- |
|  | **PWV cf, m/s** | **PWV ao, m/s** | **AIx ao, %** | **AIx br, %** | **ABI, ratio** |
| Age, yr | -0.05 (-0.14; 0.05) | -0.03 (-0.09; 0.03) | 1.84 (1.45; 2.24) | 6.60 (5.55; 7.64) | -0.008 (-0.01; -0.001) |
| Sex=female | 1.02 (0.79; 1.24) | 0.68 (0.53; 0.83) | 7.17 (6.22; 8.11) | 24.97 (22.46; 27.48) | -0.07 (-0.08; -0.05) |
| BMI, kg/m^2^ | -0.28 (-0.45; -0.10) | -0.18 (-0.30; -0.07) | -0.10 (-0.84; 0.64) | -1.48 (-3.45; 0.48) | 0.03 (0.01; 0.04) |
| Waist circumference, cm | 0.19 (-0.01; 0.40) | 0.13 (-0.008; 0.27) | -1.52 (-2.38; -0.66) | -4.95 (-7.23; -2.67) | 0.00004 (-0.01; 0.01) |
| Biking, h/week | 0.004 (-0.07; 0.08) | 0.003 (-0.05; 0.05) | 0.11 (-0.20; 0.43) | -0.03 (-0.86; 0.80) | 0.0008 (-0.004; 0.006) |
| Sports, h/week | -0.009 (-0.08; 0.07) | -0.007 (-0.06; 0.04) | -0.33 (-0.65; -0.02) | -0.84 (-1.68; -0.003) | 0.008 (0.002; 0.01) |
| Current smoker | 0.45 (0.16; 0.73) | 0.30 (0.11; 0.49) | 1.32 (0.12; 2.52) | 6.87 (3.67; 10.06) | -0.05 (-0.07; -0.03) |
| Former smoker | 0.08 (-0.08; 0.25) | 0.06 (-0.05; 0.16) | 0.68 (0.004; 1.35) | 1.55 (-0.23; 3.34) | -0.002 (-0.01; 0.009) |
| SBP, mmHg | 0.57 (0.46; 0.67) | 0.38 (0.31; 0.45) | 1.12 (0.68; 1.57) | 5.78 (4.60; 6.97) | -0.03 (-0.04; -0.02) |
| DBP, mmHg | -0.06 (-0.17; 0.05) | -0.04 (-0.11; 0.03) | 2.04 (1.59; 2.49) | 6.16 (4.97; 7.36) | -0.01 (-0.02; -0.002) |
| Pulse, beats/min | -0.17 (-0.25; -0.09) | -0.11 (-0.17; -0.06) | -2.98 (-3.32; -2.64) | -11.31 (-12.22; -10.41) | -0.01 (-0.02; -0.006) |
| Lipid-lowering drugs | -0.08 (-0.29; 0.13) | -0.05 (-0.19; 0.09) | 0.15 (-0.73; 1.02) | 0.39 (-1.93; 2.71) | -0.002 (-0.02; 0.01) |
| Antihypertensive drugs | -0.21 (-0.39; -0.04) | -0.14 (-0.26; -0.03) | -0.42 (-1.14; 0.30) | 0.16 (-1.75; 2.07) | -0.002 (-0.01; 0.01) |
| Prior CVD | 0.18 (-0.20; 0.56) | 0.11 (-0.14; 0.37) | 0.81 (-0.79; 2.41) | 1.99 (-2.25; 6.24) | -0.02 (-0.05; 0.003) |
| Total cholesterol, mmol/l | -0.15 (-0.40; 0.10) | -0.10 (-0.27; 0.07) | -0.90 (-1.95; 0.15) | -2.81 (-5.59; -0.04) | 0.05 (0.03; 0.06) |
| HDL-cholesterol, mmol/l | 0.10 (-0.04; 0.23) | 0.06 (-0.02; 0.15) | 0.54 (-0.02; 1.09) | 1.72 (0.24; 3.19) | -0.02 (-0.03; -0.01) |
| LDL-cholesterol, mmol/l | 0.06 (-0.16; 0.29) | 0.04 (-0.11; 0.20) | 0.72 (-0.24; 1.67) | 2.00 (-0.52; 4.51) | -0.03 (-0.05; -0.02) |
| Triglycerides (log10) | 0.11 (0.003; 0.22) | 0.07 (0.001; 0.14) | 0.26 (-0.19; 0.71) | 1.00 (-0.19; 2.19) | -0.02 (-0.02; -0.009) |
| CRP (log10) | -0.02 (-0.10; 0.06) | -0.01 (-0.07; 0.04) | 0.19 (-0.16; 0.53) | 0.84 (-0.07; 1.75) | -0.005 (-0.01; 0.0005) |
| HbA_1c_ (log10) | 0.09 (0.0005; 0.17) | 0.06 (0.001; 0.11) | -0.25 (-0.60; 0.11) | -1.56 (-2.50; -0.62) | 0.002 (-0.004; 0.008) |
| Skin AF, arbitrary units | 0.19 (0.11; 0.27) | 0.13 (0.07; 0.18) | 0.49 (0.14; 0.84) | 2.04 (1.11; 2.97) | -0.02 (-0.02; -0.01) |

N=3535. Continuous variables were standardized to compare effect estimates on the same scale. AF=autofluorescence, EPIC-DZD=Sub-study of European Prospective Investigations into Cancer and Nutrition, SBP=systolic blood pressure, DBP=diastolic blood pressure, CVD=cardiovascular disease, HDL=high density lipoprtotein, LDL=low density lipoprotein, CRP=C-reactive protein, HbA_1c_=glycated hemoglobin 1C.
